# High-performance flexible organic field effect transistors with print-based nanowires

**DOI:** 10.1038/s41378-023-00551-x

**Published:** 2023-06-13

**Authors:** Liangkun Lu, Dazhi Wang, Changchang Pu, Yanyan Cao, Yikang Li, Pengfei Xu, Xiangji Chen, Chang Liu, Shiwen Liang, Liujia Suo, Yan Cui, Zhiyuan Zhao, Yunlong Guo, Junsheng Liang, Yunqi Liu

**Affiliations:** 1 Laboratory for Micro/Nano Technology and System of Liaoning Province, Dalian University of Technology, Dalian, 116024 China; 2 State Key Laboratory of High-Performance Precision Manufacturing, Dalian University of Technology, Dalian, China; 3 Ningbo Institute of Dalian University of Technology, Ningbo, 315000 China; 4 Beijing National Laboratory for Molecular Sciences, Key Laboratory of Organic Solids, Institute of Chemistry, Chinese Academy of Sciences, Beijing, 100190 China

**Keywords:** Electronic devices, Electrical and electronic engineering

## Abstract

Polymer nanowire (NW) organic field-effect transistors (OFETs) integrated on highly aligned large-area flexible substrates are candidate structures for the development of high-performance flexible electronics. This work presents a universal technique, coaxial focused electrohydrodynamic jet (CFEJ) printing technology, to fabricate highly aligned 90-nm-diameter polymer arrays. This method allows for the preparation of uniformly shaped and precisely positioned nanowires directly on flexible substrates without transfer, thus ensuring their electrical properties. Using indacenodithiophene-co-benzothiadiazole (IDT-BT) and poly(9,9-dioctylfluorene-co-benzothiadiazole) (F8-BT) as example materials, 5 cm^2^ arrays were prepared with only minute size variations, which is extremely difficult to do using previously reported methods. According to 2D-GIXRD analysis, the molecules inside the nanowires mainly adopted face-on π-stacking crystallite arrangements. This is quite different from the mixed arrangement of thin films. Nanowire-based OFETs exhibited a high average hole mobility of 1.1 cm^2^ V^−1^ s^−1^ and good device uniformity, indicating the applicability of CFEJ printing as a potential batch manufacturing and integration process for high-performance, scalable polymer nanowire-based OFET circuits. This technique can be used to fabricate various polymer arrays, enabling the use of organic polymer semiconductors in large-area, high-performance electronic devices and providing a new path for the fabrication of flexible displays and wearable electronics in the future.

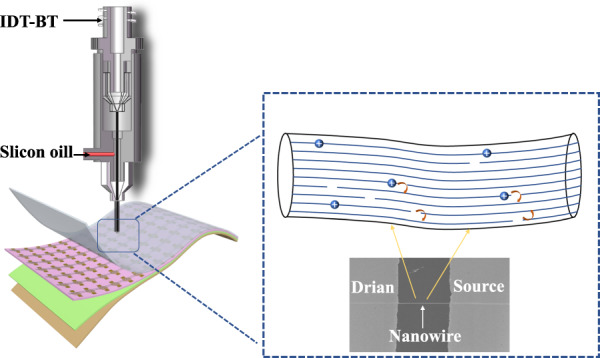

## Introduction

Flexible electronics have attracted considerable attention because of their advantages of portability, folding capabilities and scalability, which also show great potential in the development of organic electronics^1–4^, including wearable and smart electronic devices ranging from electronic skin and artificial intelligence technology to flexible displays^5–10^. Usually, organic films prepared by spin coating or solution shearing approaches have been widely used to fabricate organic field-effect transistors (OFETs)^11–17^. However, the mobility of these OFETs remains at a relatively low level compared to inorganic devices and exhibits complex preparation processes, such as multistep lithography and transfers^14,18,19^. In addition, the organic films prepared via these methods are generally characterized by nonuniform crystal morphology and growth direction, which cause considerable limitations in their electronic properties and greatly hinder practical applications.

In recent years, the application of one-dimensional (1D) semiconductor micro/nanowires in OFETs has been studied^20–26^. Wang chose cyclopentadithiophene-benzothiadiazole copolymer (CDT-BTZ)-fabricated polymer microwires for OFETs, and the results showed that the self-assembled microwires in the molecular chains were highly oriented in the direction of charge carrier transport, with mobilities as high as 5.5 cm^2^ V^−1^ s^−1^, and the width of the microwires ranged from 0.3 μm to 0.6 μm with lengths from 5 to 20 μm^26^. Wu et al. proposed a novel capillary bridge self-assembly strategy to prepare polymer microwires, and their results showed that the hole mobility of microwire-based OFETs was 6.8 × 10^–3^ cm^2^ V^−1^ s^−1^, which was 3× higher than that of thin films^27^. However, the micro/nanowires fabricated above only handled the problem of preparation of OFETs on rigid substrates, meaning that additional fabrication processes such as transfer will be required for the realization of flexible devices with micro/nanowires. Furthermore, the large number of micro/nanowire alignments with high resolution is a significant limitation for the above fabrication methods (such as capillary bridges and solvent vapor-enhanced drop casting). Thus, this represents a significant bottleneck in the development OFETs for further performance improvement and wide applications^28,29^. As a consequence, the technology for fabricating large-area and controllable organic semiconductor micro/nanowires on flexible substrates still remains to be established, and high-performance flexible OFETs with highly aligned micro/nanowires need to be developed.

A technique called coaxial focused electrohydrodynamic jet (CFEJ) printing was proposed in this work, which provides a novel method for producing large-area, controllable organic polymer semiconductor nanowires (NW) on flexible substrates. NWs with a width of 90 nm were achieved on 5 × 5 cm^2^ flexible substrates. In addition, the pattern of curbing and crossing structures was also printed with high resolution (300 nm). According to two-dimensional grazing-incidence X-ray diffraction (2D-GIXRD) results, the molecules inside the nanowires mainly adopted face-on π-stacking crystallite arrangements, and the π–π stacking distance (d_π–π_) was 4.23 Å, which is quite different from the mixed arrangement of thin films. Furthermore, print-based OFETs were realized, which show a mobility of 1.1 cm^2^ V^−1^ s^−1^, which is 5× higher than that of conventional thin film-based OFETs under the same conditions, and their on/off ratio was 1.93 × 10^5^. We believe that the NW structures fabricated using CFEJ printing technology and NW-based OFETs can open up a new way for the preparation of large-area and high-performance displays, wearable electronics and flexible sensors.

## Results

### Experimental setup and working principle

**Fig. 1:**
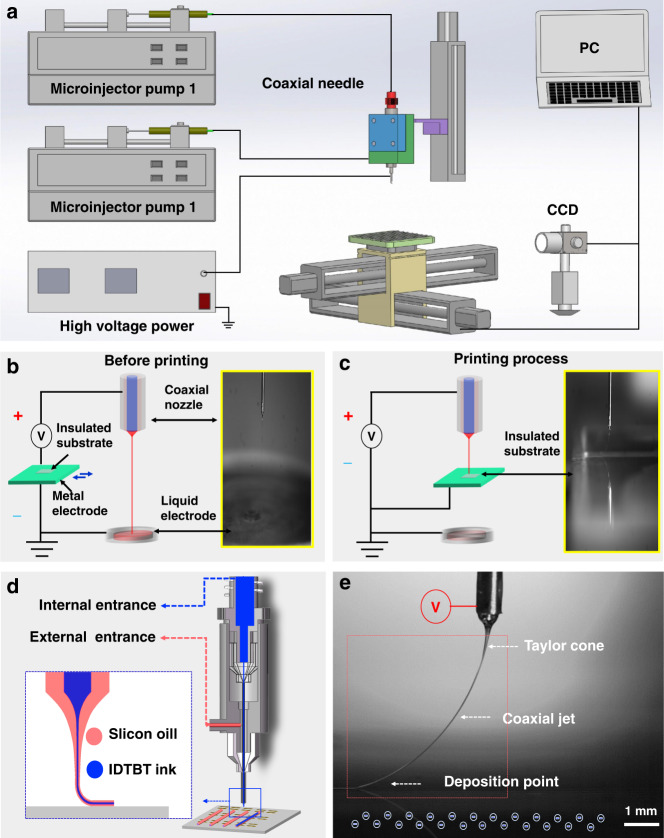
The CFEJ printing equipment and processes. **a** Diagram of the composition of the CFEJ printing system. **b** Schematic of the liquid–metal double electrode structure. **c** Processing of the liquid–metal double electrode structure, where the illustration shows wrapping of the structures on the insulating substrate. **d** Schematic of the coaxial nozzle. **e** Single frame captured from the video recording of the process to enable analysis of the process signatures of CFEJ printing

The CFEJ printing system comprises a coaxial needle, two microinjector pumps, a high-voltage power supply, and a three-axis movement stage (Fig. 1a). The coaxial nozzle consists of two coaxially located inner and outer nozzles. In the system, microinjector pump 1 is connected to the inner nozzle, and microinjector pump 2 is connected to the outer nozzle. IDT-BT ink and silicone oil are delivered simultaneously to the coaxial needle through the inner and outer needles and extruded at the tip of the needle under the push of the microinjector pumps. The liquid at the tip of the needle will form a Taylor cone, and when the voltage continues to increase, a coaxial jet will be generated. In conventional E-jet printing processes, ink materials are directly printed on the grounding electrode. The printed materials carry residual charge, and the residual charge density increases with the accumulation of ink on the grounding electrode. The polarity of the residual charge is accordingly with the coaxial jet, and a Coulomb repulsion force will be generated between the printed ink materials and coaxial jet^30–32^. This Coulomb repulsion force will cause the coaxial jet to become unstable and result in the whipping phenomenon. In addition, when the residual charge density increases, the whipping phenomenon becomes serious. Subsequently, this will lower the stability and repeatability of the CFEJ printing process (Supplementary movie 1). In this work, a strategy of using metal-liquid composite electrodes is proposed (Fig. 1b, c). Approximately 10 ml of solvent was added to the liquid electrode tank (a mixed solvent of isopropanol and cyclohexane was used in this work). Before the printing process begins, the coaxial jet flowing from the coaxial nozzle will first enter the liquid electrode, dissolving the outer silicon oil and the IDT-BT ink and neutralizing the charge. Simultaneously, the heating plate located in the bottom of the liquid electrode tank heats the mixed solvent to 40 °C, thus improving the dissolution rate of the coaxial jet (inset of Fig. 1b and Supplementary movie 2). During the printing process, the coaxial jet initially contacts the liquid electrode before it reaches the metal moving substrate, and the outer wrapped material is dissolved rapidly, which avoids the charge accumulation problem caused by material accumulation and eliminates the jet whip caused by Coulomb repulsion. Therefore, these metal–liquid composite electrodes help increase the coaxial jet’s stability as well as the uniformity of the printed structure during the process (Fig. 1c and Supplementary Fig. 1).

While applying voltage between the coaxial nozzle and the metal–liquid composite electrodes, a polarized charge will be generated inside the liquid, and the polarized charge and the free charge in the liquid will move toward the outer surface of the liquid under the action of the electric field. The charge gathered on the surface of the liquid produces an electric field force and drives the liquid to focus inward. Since the external silicone oil is an insulating material (the dielectric constant is 2.77), the surface charge formed by the applied voltage exists only at the interface between the interior IDT-BT ink and the exterior silicone oil, as well as at the interface between the air and the silicone oil. The surface charge transfer on the outer surface of the inner IDT-BT ink and outer silicone oil will result in electrical shear forces acting on both the inner IDT-BT ink and the silicone oil. The inner electric shear force acts directly on the inner IDT-BT ink, making the IDT-BT ink jet diameter substantially shrink. At the same time, the electrical shear force generated by the high viscosity silicone oil is also transferred to the internal IDT-BT ink and results in a reduction in the internal jet diameter. In addition, the external silicone oil can prevent the IDT-BT ink from interference by airflow, vibration and temperature (Fig. 1d). This home-built printing equipment achieved all printing processes carried out in air, and user-friendly control software for this equipment was also developed, which has the potential to be used in commercial production.

### Large-area NW arrays on flexible substrates by CFEJ printing

**Fig. 2:**
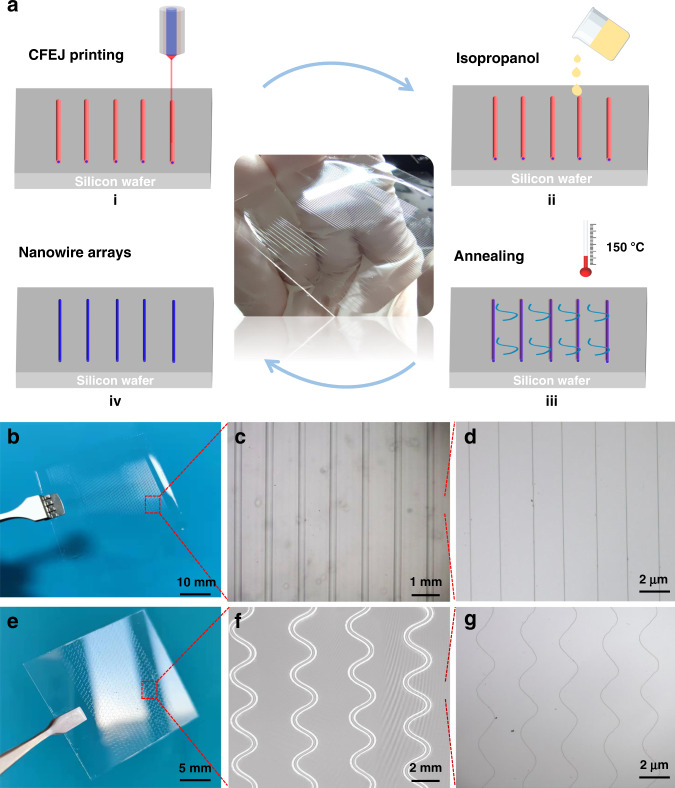
Flow diagram for the removal of silicon oil and structures fabricated on flexible substrates. **a** Flow diagram for removal of the outer high-viscosity silicone oil by the solution method: (i) printed wrapped structures; (ii) placement of the printed sample in isopropanol at 70 °C to remove the outer silicone oil coating; (iii) evaporation of the residual solvents and annealing of the polymer, where the temperature is 150 °C; (iv) inner polymer NW arrays obtained after removal of the outer silicone oil. **b**–**d** Optical images of NW arrays on PDMS substrate. **e**–**g** Optical images of sinuous structures on PET substrate

As depicted schematically in Fig. 2a, IDT-BT ink/silicone oil bilayer structures were fabricated by CFEJ printing technology on flexible substrates (Fig. 2a–i). Then, the wrapped structures were placed in an isopropanol solution and heated to 70 °C to dissolve the outer silicone oil (Fig. 2a-ii). Subsequently, the printed sample was placed on a 150 °C heating plate to evaporate the residual isopropanol completely (Fig. 2a-iii). Finally, IDT-BT NWs were obtained (Fig. 2a-iv). Mass preparation of controllable NW structures on flexible substrates is one of the keys to achieving high-performance organic electronics. In this work, linear and sinuous arrays of NW structures were successfully prepared on polydimethylsiloxane (PDMS) and polyethylene terephthalate (PET) substrates using the CFEJ printing technique. To fabricate linear structures, the concentration of the IDT-BT ink is 6 mg mL^−1^, and the printing parameters of working distance, applied voltage, silicone oil flow rate, IDT-BT ink flow rate, and printing speed are 3 mm, 4.5 kV, 4 µL min^−1^, 100 nL min^−1^ and 350 mm s^−1^, respectively^27^. With the use of these parameters, the IDT-BT NW arrays were produced on the PDMS flexible substrate, as shown in Fig. 2b and Supplementary Movie 3. The results showed that the alignment of the IDT-BT ink/silicone oil wrapped structure was very good. The application of liquid electrodes in this large area-controlled printing process, which can prevent the accumulation of charge, avoids the jet whipping issue that often occurs in the conventional printing method.

In recent years, implantable sensors and printing flexible electronics have been widely used. However, patterning polymer semiconductors with high resolution on flexible substrates remains a challenge. With conventional methods for printing on insulating substrates, although Taylor cones can be formed at the tip of the nozzle, the presence of evanescent electric field forces prevents the Taylor cones from continuing to develop and thus cannot be printed on flexible substrates. It is worth mentioning that large-area, high-resolution polymer nanostructure preparation on flexible substrates was achieved in this work, and the printing maximum array can reach 8 inches. This is mainly due to the use of metal-liquid composite electrodes to avoid charge accumulation and Coulomb repulsion. Moreover, the working distance was raised to 4 mm by reducing the printing speed to 180 mm, and other parameters were kept constant. Sinuous structures with high similarity were obtained on PET substrates (Fig. 2e and Supplementary movie 4). As indicated by Fig. 2f, IDT-BT ink/silicone oil bilayer structures printed on flexible substrates exhibit a high degree of self-similarity and controllability. After the external silicone oil was removed, sinuous NWs were obtained (Fig. 2g), which can greatly increase the stretchability of the OFETs and thus offer great application potential for OFETs in flexible devices and wearable devices.

**Fig. 3:**
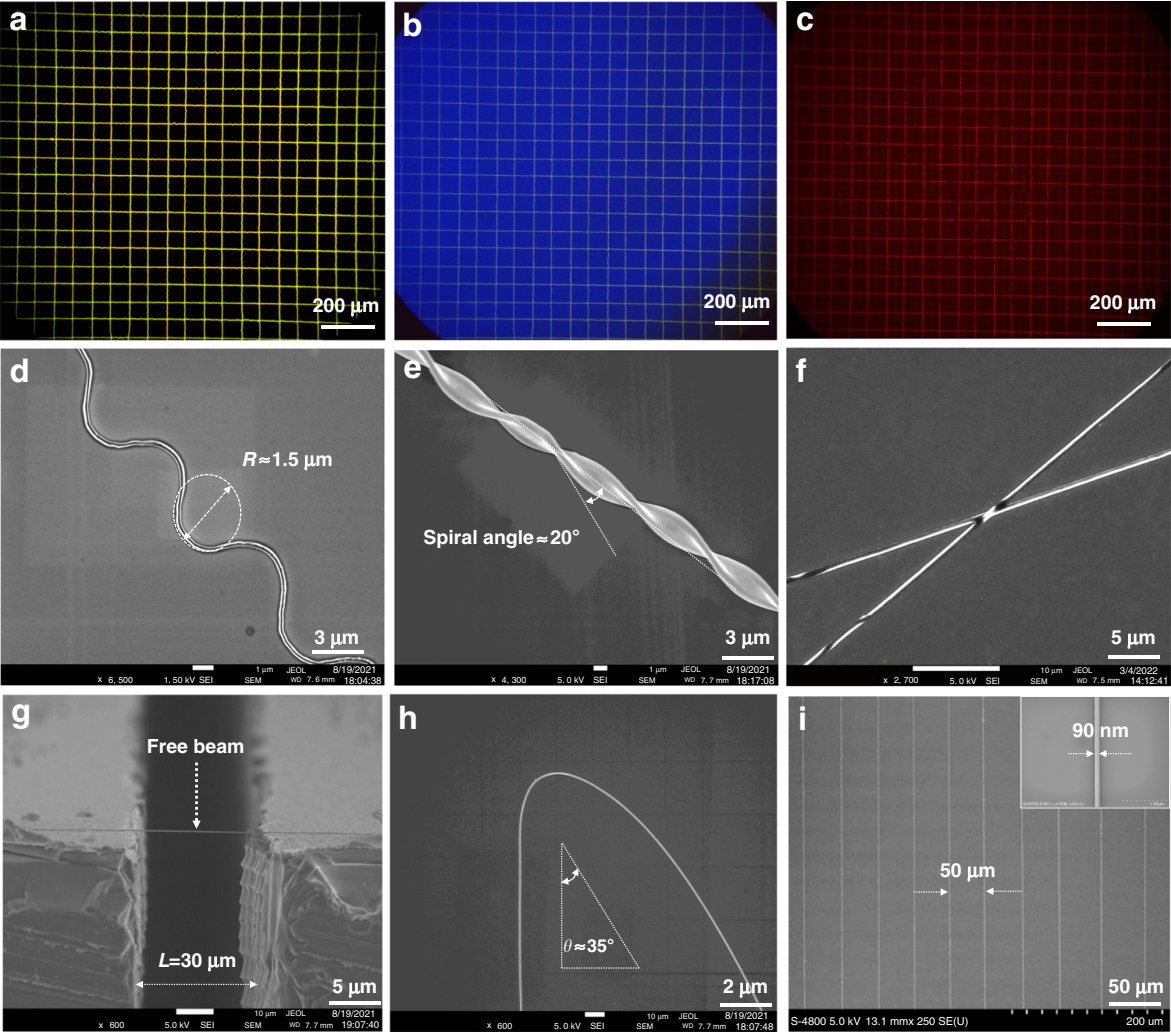
Scanning electron microscope (SEM) images of different structures fabricated using CFEJ printing technology. **a**–**c** Large array network structure composed of F8-BT ink. **d** IDT-BT sinuous structure with a bending radius of ~1.5 μm. **e** IDT-BT helical structure with a width of ~3 μm. **f** IDT-BT crossed structure with a width of ~300 nm. **g** IDT-BT nanoscale free beam array with a width of ~120 nm. **h** Curved line structure with a width of ∼100 nm. **i** IDT-BT NW array with a width of ∼90 nm

Figure 3 shows various organic semiconductor patterns that were printed using the CFEJ printing technique. Figure 3a–c shows the printed network structure with poly(9,9-dioctylfluorene-co-benzothiadiazole) (F8-BT) ink, along with fluorescence microscopic images acquired under irradiation by blue, red, and green colored lights. Figure 3d shows the printed wriggle structure, where the radius of curvature of the IDT-BT wriggle structure was 1.5 µm, showing that the CFEJ printing technology can be utilized to fabricate flexible electronics and improve their tensile qualities. During the process of removing the outer layer of silicon oil, a very thin probe was used to fix one end of the printed NW, and then the probe was rotated, which drove the NW in a spiral motion. After waiting for the outer layer of silicon oil to completely dissolve in the isopropanol solution, the helical structure can be obtained, as shown in Fig. 3e, thus illustrating the potential for the applications of artificial muscles and wearable sensors. Figure 3f shows the crossed IDT-BT NWs with a width of ∼300 nm. The image shows good contact and clear boundaries between the nanowires at the intersections, which demonstrates the potential of CFEJ printing technology for fabricating multilayer and three-dimensional structures. Figure 3g shows a printed IDT-BT nanoscale free beam, which shows straight and homogeneous characteristics crossing the gap. Compared to NW structures constrained to a substrate, nanobeam structures show better properties, such as a larger specific surface area and larger vibration amplitude, due to the removal of substrate constraints. This provides more significant advantages in high-performance nanodevices and shows the advantages of CFEJ printing technology in the fabrication of special structures. Figure 3h depicts the printed curved line structures, which are useful in folding devices. The width of NWs can be further reduced by increasing both the printing speed and applied voltage while simultaneously decreasing the needle-to-substrate distance and the flow rate of IDT-BT ink. Therefore, the printing speed is increased to 400 mm s^−1,^ and the voltage is increased to 6 kV during the printing process. The results indicate that printed IDT-BT NWs with a width of 90 nm were obtained (Fig. 3i). Compared with others, the nanowires prepared in this work achieved reproducibility, controllability and high alignment^21,27,33^. The manufacture of IDT-BT nanostructures with these dimensions using typical manufacturing methods would require the use of high-precision equipment for accurate alignment, which is expensive, and the entire fabrication process would be difficult and time-consuming. The proposed CFEJ printing technology realized the manufacture of these nanostructures with a homogeneous and smooth configuration in a single step. In addition to IDT-BT and F8-BT, other polymer semiconductor materials, including N2200, P3HT and PDVT-10, were also employed for pattering structures using the CFEJ printing technique (Supplementary Fig. 2), which also showed smooth and highly aligned morphologies. This result exhibits the wide material application ranges of CFEJ printing technology. CFEJ printing technology enables the printing of a wide range of materials, such as metals, piezoelectric materials, insulation materials and semiconductors. Generally, materials that meet a suitable viscosity range of 1–10,000 cps can be printed with CFEJ printing technology.

### Morphology and crystallinity analysis of polymer NWs

It is well known that the morphology and crystallinity of organic semiconductors have an important influence on the performance of organic electronics. Atomic force microscopy (AFM) was used to study the morphology of the NWs prepared by CFEJ printing and the thin films prepared by spin-coating. AFM images (Supplementary Fig. 3) showed that the NWs have a uniform width and no obvious grain boundaries or defects. In contrast, the film prepared by the spin-coating method has obvious defects, and its surface is rough. The different morphologies of printed NWs and spin-coated films will cause differences in the carrier transport efficiency. This difference is mainly due to the polymer being wrapped inside the silicone oil after the printing process, and the silicone oil produces a uniform shrinkage force and internal pressure on the internal IDT-BT polymer, and these forces are greatly beneficial to the formation of a smoother morphology. Since the silicone oil is wrapped around the IDT-BT ink, the volatilization of the IDT-BT ink solvent requires a long annealing process (~600 min), and the very slow volatilization of the solvent during this process also promotes the formation of a smoother surface.

**Fig. 4:**
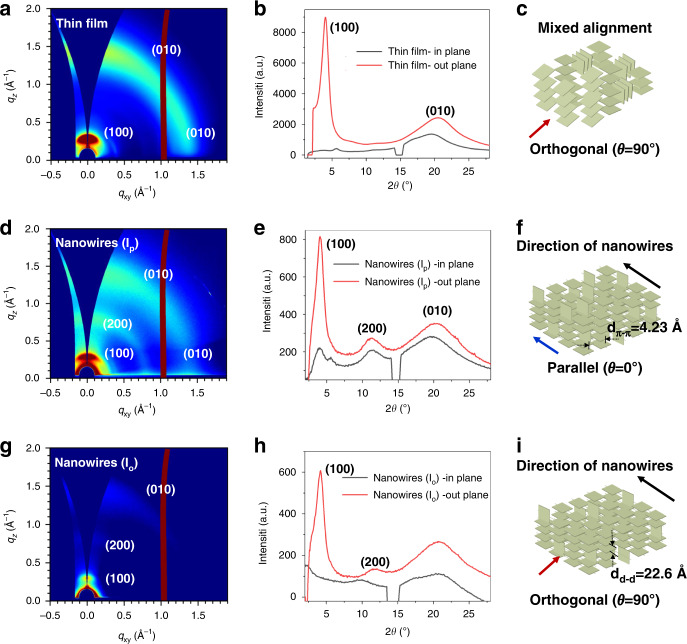
GIXRD analysis of IDT-BT NWs and thin film. **a**, **b** GIXRD analysis of the thin film. **c** Diagram of the mixed arrangement in the IDT-BT thin film. **d**, **e** GIXRD analysis of NWs (the direction of the light is the same as the NWs). **f** Diagram of the face-on arrangement in NWs. The blue arrow in the bottom left indicates that the direction of the light is the same as that of the nanowires. **g**, **h** GIXRD analysis of NWs (the direction of the light is perpendicular to the NWs). i) Diagram of π–π stacking in NWs. The red arrow in the bottom left indicates that the direction of the light is perpendicular to the NWs

Furthermore, 2D-GIXRD is a powerful method to analyze the structural information of materials, such as crystallization orientation and π-stacking distance^34–37^. Then, 2D-GIXRD analysis was conducted for printed IDT-BT NWs and spin-coated thin films. In the 2D-GIXRD results, a ring of uniform intensity represents a poor orientation, while a speckle or arc represents a selective orientation^38,39^, and the correspondence between diffraction peaks and texture is shown in Supplementary Fig. 4. Figure 4a, b indicates that there are (010) diffraction peaks in both the in-plane and out-of-plane, as well as one (100) diffraction peak in the out-of-plane. This indicates that both edge-on and face-on arrangements exist inside the film and that the probabilities of both arrangements are at the same level. This means that the crystallinity of the IDT-BT thin film was low, and the stacking orientation was negative (Fig. 4c). However, in the results of printed NWs, two clear diffraction peaks of (100) and (200) were generated (Fig. 4d, e). The incident direction of the light was set parallel (I_p_) to the direction of the NWs. Compared to the thin film, it is obvious that there is one more diffraction peak than the thin film, which indicates that the printed NWs have enhanced orientation and an increased proportion of internal stacking by face-on (Fig. 4f). This can effectively reduce carrier trapping and improve carrier mobilities. Meanwhile, it was found that the intensity of the (010) diffraction peak is much weaker than that of the film, which demonstrates that the polymer molecular chains inside the NWs mainly exhibit face-on and parallel arrangements. The π-stacking direction (010) of the majority crystalline is orthogonal to the direction of the NWs, and a π–π stacking distance (d_π–π_) of 4.23 Å was calculated by the equation *d* = *λ/*2 sin *θ*^13^. When the incident direction of the light is orthogonal (I_o_) to the direction of the NWs, the (010) signal is not visible (Fig. 4g), indicating that the polymer molecular chains are aligned orthogonal to the direction of incident light. Figure 4h shows the NWs’ in-plane and out-of-plane 1-D GIXRD measurements. The (010) signal was only apparent when the incident light direction was parallel to the NWs, indicating that the molecular chains within the NWs were mostly arranged face-on and parallel to the direction of the NWs (Fig. 4i).

Supplementary Fig. 5 shows the 2D-GIXRD characterization results for samples printed with various numbers of NWs. When the number of NWs is below 2000, no diffraction signal is observed; when the number of NWs increases to 4000, two weak diffraction peaks of (100) and (200) can be seen. When the number of NWs increased to 5000, two clear diffraction peaks of (100) and (200) were generated. The (010) signal was only apparent when the incident light direction was parallel to the NWs. This indicates that the diffraction signal is more distinct as the number of NWs increases, also indicating that the molecular chains within the NWs are arranged.

This difference is primarily due to the high-speed drag behavior of the CFEJ printing process, which may promote molecular chain alignment inside the polymer NWs. This variation is primarily due to the high-speed drag behavior of the CFEJ printing process, which may promote molecular chain alignment inside the polymer NWs. This is mainly due to the high viscosity of the external silicone oil (6 × 10^4^ cst), a mechanical drag on the coaxial jet and the needle-substrate will be generated when the high-speed moving substrate (400 mm s^−1^) contacts the coaxial jet, and the drag force is consistent with the printing direction. Therefore, it is possible that this drag force promotes the orderly alignment of polymer molecular chains in the same direction as the NWs.

### High-performance print-NW-based flexible OFETs

**Fig. 5:**
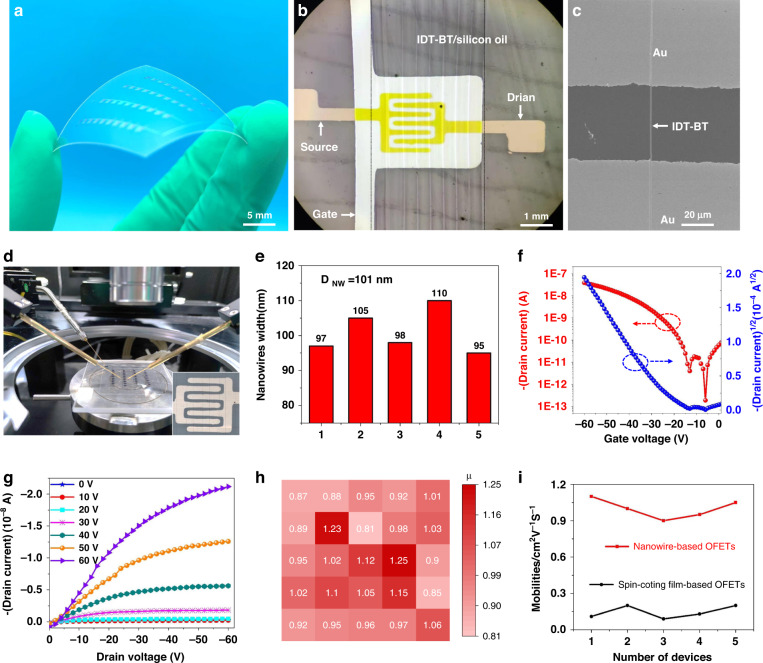
Device performance characteristics of IDT-BT NW-based OFETs. **a** Photograph of a 4 × 10 OFET array on a flexible PET substrate. **b** Optical microscope image of the OFET array. **c** SEM image of a single nanowire. **d** Test platform for the polymer NW-based OFETs. **e** IDT-BT NW diameter statistics. **f**, **g** Transfer and output characteristics of NW-based OFETs. **h** Distribution of mobilities measured from a 4 × 10 array of OFETs. **i** Comparison of mobilities between NW-based OFETs and thin film-based OFETs

Flexible OFET devices of 4 × 10 arrays were fabricated on 100-μm-thick PET films. Supplementary Fig. 6a and b shows the schematic of OFETs with bottom-gate and bottom-contact structures and the preparation process of the flexible NW-based OFETs. Figure 5a–c and Supplementary Fig. 7 show NW-based OFETs containing detailed IDT-BT nanowires. The NW arrays are highly ordered and have good contact with the source-drain electrodes through the channels. Figure 5d provides an optical image of the fabricated NW-based flexible OFETs on the testing platform, where the channel length is ∼50 μm. The NWs in the flexible OFET have a uniform width of *D*_NW_ = 101 nm (Fig. 5e). The typical p-type transfer and output characteristics of the IDT-BT NW-based flexible OFETs are shown in Fig. 5f, g. The results show that almost no hysteresis is observed in the forward and backward scans, along with a high current on-off (*I*_on_/*I*_off_) ratio (≈1.93 × 10^5^). The field-effect hole mobility of the NW-based OFETs was calculated to be 1.1 cm^2^ V^−1^ s^−1^ using the formula 2d*I*_ds_/d*V*_*gs*_ *L* = *µ W C*_*i*_, where *µ* is the mobility of the OFET, L is the length of the source-drain channel, W is the sum of the widths of the printed NWs, K is calculated from the ratio of *V*_Gs_ and $\surd {I}_{{DS}}$, and C is the dielectric constant of PMMA^27,40,41^. Figure 5h shows the statistical mobility of the devices. Each square represents an OFET device, where the color intensity represents the measured performance in each case. It can be seen in the graph that the highest mobility of the devices can reach 1.25 cm^2^ V^−1^ s^−1^, and all devices in these arrays can work effectively and show consistent performance. The mobility of NW-based OFETs prepared by CFEJ printing technology was five times higher than that of thin film-based OFETs (0.2 cm^2^ V^−1^ s^−1^) prepared by spin coating, as shown in Supplementary Fig. 8. The reason is mainly due to the extremely regular molecular arrangement of the polymer NWs, which is similar to that of a single crystal. In conventional thin-film-based OFETs, carriers are transported mainly in the π–π direction. It can be inferred from the GIXRD results that the carrier transport in NW-based OFETs is a combination of main chain transport and π–π directional transport. First, the carriers are mainly transported along the direction of the main chain, and when the carriers reach the end of the main chain, they jump to another chain via π-π transport and continue along the main chain. Since the main chain is aligned in the same direction as the nanowires, the carrier transport efficiency is more efficient than that of the thin film. In addition, the diameter of NWs is much smaller than that of thin films, which results in a much lower contact resistance between the NWs and the source-drain electrodes, which also improves the performance of the OFETs. When compared with the OFETs reported previously in the literature^2,22,28,42^, as summarized in Table 1, the polymer NW-based OFETs reported in this work can achieve a resolution of 90 nm with a smooth morphology while also achieving a hole mobility of ≈1.1 cm^2^ V^−1^ s^−1^ with a high *I*_on_/*I*_off_ ratio of ≈1.93 × 10^5^. More importantly, these polymer NW structures can be manufactured at ambient temperature and with high controllability, thus making them suitable for scalable device manufacturing, transfer, and integration onto soft elastomer substrates. When combined, these qualities form a solid foundation for the investigation of a new technological practice for flexible electronics integration with the aim of realizing a broader variety of soft electronic device applications.

**Table 1:** Comparison of IDT-BT NW-based OFETs on PET with other OFET devices in the literature

Channel materials	Fabrication techniques	Substrates	Dnw (nm)	Mobility [cm^2^V^−1^s^−1^]	Refs.
TIPS-TAP	Channel-restricted and transfer	Glass	550 ~ 600	0.98	2
PEDOT NWs	Direct printing and vaporphase polymerization	Si	90	0.15	22
P4TDPP NWs	Electrospun	Si	190	0.305	28
P3HT NWs	Electrospun	Si	180	0.03	42
IDT-BT NWs	Direct printing	PET	90	1.25max	This work

## Conclusion

In summary, we have developed a universal CFEJ printing method that can be used to fabricate highly aligned polymer arrays at RT without photolithography or transfer processes. Large-area production of polymer NWs with the same geometries is possible using the proposed technique. CFEJ printing technology offers the advantages of simple operation, high resolution, and material savings and can achieve highly aligned, controllable, and efficient fabrication of IDT-BT polymer NW array structures. The length of a single NW exceeds 50 mm, and the NW arrays have a uniform width that can be stabilized at ~90 nm. According to 2D-GIXRD analysis, the molecules inside the nanowires mainly adopted face-on π-stacking crystallite arrangements. This is quite different from the mixed arrangement of thin films. Nanowire-based OFETs showed a high average mobility of 1.1 cm^2^ V^−1^ s^−1^ and good device uniformity, which is 5× that of thin film-based OFETs fabricated under the same conditions. In addition, the on/off ratio of the OFETs was 1.93 × 10^5^. NW arrays prepared by CFEJ printing provide a simple way to fabricate high-performance OFETs. This method provides a possible route toward the realization of highly uniform polymer arrays with high intrinsic mobility.

## Methods

### Materials

IDT-BT was prepared according to previous procedures reported in the literature and had an average molecular weight of 47.5 kDa. To fabricate the polymer ink, the IDT-BT solute was first heated at 80 °C for ~60 min to ensure the drying of the solute. After that, the IDT-BT solvent was mixed with o-xylene for almost 300 min while being heated to 80 °C. Similarly, F8-BT ink was prepared by adding F8-BT solute with a concentration of 5 mg ml^−1^ to a chlorobenzene solvent and magnetically stirring the mixture at 80 °C for ~300 min. The viscosity of the silicone oil used in this study was 6 × 10^4^ cst, and the dielectric constant was 2.77. Silicone oil was also purchased from Sigma‒Aldrich and did not need to be purified.

### Experimental setup of coaxial needle

The coaxial needle is composed of internal and external stainless-steel needles, which need to be installed with a high degree of coaxiality (<10 μm). The inner needle contained polymer ink connected to microinjector pump 1 (Harvard Apparatus, Holliston, MA, USA) with a supply rate of 30–200 nL min^−1^. The outer needle contained silicone oil connected to microinjector pump 2 with a supply rate of 1–10 μL min^−1^. The diameters of the inner and outer needles are 110 μm and 460 μm, respectively. The external nozzle is connected to a supply power (Dongwen, Tianjin, China) with an operating voltage of 3.5–6.5 kV. The applied voltage creates a stable electric field between the coaxial needle and the substrate. The substrate is grounded and mounted on a computer-controlled 3-axis motion stage (HIWIN, Taiwan). A color camera was used to observe the morphology of the coaxial jet during the printing process (CM3-U3–50S5C-CS, FLIR, USA). A high-speed framing camera was utilized to capture the coaxial jet being collected on the substrate (CAMCUBE7, Mikrotron GmbH, Germany).

### Fabrication process and evaluation of OFETs

The OFET devices were fabricated with a top contact configuration. First, 200-µm-thick PET films were used as flexible substrates, and each substrate was first annealed using steps of 100, 130, and 160 °C with a treatment time interval of 30 min to release any thermal stress. The substrate was then placed into isopropyl alcohol and ultrasonically cleaned at 100 W for 10 min. Then, a 100-nm-thick Al film was vaporized to act as the gate electrode layer, and an 800-nm-thick PMMA film was subsequently spin coated to act as the dielectric layer (PMMA dissolved in n-butyl acetate, with a curing agent ratio of 10:6 by mass). The relative dielectric constant of this dielectric material is 2.8, which is equivalent to a capacitance. surface morphology of PMMA was investigated, and the root-mean-square (RMS) roughness values of PMMA were found to be 0.7 nm (Supplementary Fig. 9), which lays the foundation for highly aligned nanowire printing. After spin-coating and annealing at 60 °C for 30 min, a 30 nm Au film was vaporized to act as the source–drain electrode layer, and the channel length was ~50 µm. The IDT-BT polymer was printed on the source–drain electrodes using CFEJ printing patterning equipment. During the printing process, the temperatures of the insulating substrate and the nozzle were maintained at 30 °C. After the printing process, the flexible substrate was placed on a hot plate at 100 °C for 8 h to allow the polymer ink to crystallize and fully contact the electrodes. The substrate was then placed in a Petri dish containing isopropyl alcohol and heated to 70 °C to dissolve the outer silicone oil layer (Fig. 2a-iii); it was then removed after 15 min and annealed at 150 °C for 10 min, and the patterned semiconductor layer was spin-coated with a PDMS layer to act as an encapsulation layer to ensure the stability and enhance the lifetime of the device. A semiconductor analyzer was used to measure the transfer and output curves (4200-SCS, Keithley, USA), and all tests were performed in air at room temperature. A scanning electron microscope (SU8220, Hitachi, Japan) was used to obtain microscopic images at accelerating voltages of 5–15 kV. One-dimensional, two-dimensional and three-dimensional images of the surface morphology were obtained using an atomic force microscope (Nanowizard 4XP, Bruker, Germany) in tap mode.

## Supplementary information

Revised Supplementary figures

Supplementary movie-1

Supplementary movie-2

Supplementary movie-3

Supplementary movie-4

## Data Availability

The data that support the findings of this study are available from the corresponding author upon reasonable request.

## References

[1] Deng W (2021). Water-surface drag coating: a new route toward high-quality conjugated small-molecule thin films with enhanced charge transport properties. Adv. Mater..

[2] Deng W (2019). Channel-restricted meniscus self-assembly for uniformly aligned growth of single-crystal arrays of organic semiconductors. Mater. Today.

[3] Song X (2022). Highly stretchable high-performance silicon nanowire field effect transistors integrated on elastomer substrates. Adv. Sci..

[4] Chen H (2012). Highly pi-extended copolymers with diketopyrrolopyrrole moieties for high-performance field-effect transistors. Adv. Mater..

[5] Ding B (2010). Electrospun nanomaterials for ultrasensitive sensors. Mater. Today.

[6] Hassan, M. et al. Significance of flexible substrates for wearable and implantable devices: recent advances and perspectives. *Adv. Mater. Technol.* **7** (2021).

[7] Shaymurat T (2013). Gas dielectric transistor of CuPc single crystalline nanowire for SO(2) detection down to sub-ppm levels at room temperature. Adv. Mater..

[8] An HS (2019). High-resolution 3D printing of freeform, transparent displays in ambient air. Adv. Sci..

[9] Cai L (2018). Direct printing for additive patterning of silver nanowires for stretchable sensor and display applications. Adv. Mater. Technol..

[10] Chen J (2022). Flexible ionic-gel strain sensor with double network, high conductivity and high frost-resistance using electrohydrodynamic printing method. Addit. Manuf..

[11] Wang J, Gu J, Zenhausern F, Sirringhaus H (2006). Low-cost fabrication of submicron all polymer field effect transistors. Appl. Phys. Lett..

[12] Wang, S. et al. Microribbon field-effect transistors based on dithieno[2,3-d;2,3’-d’]benzo[1,2-b;4,5-b’]dithiophene processed by solvent vapor diffusion. **23**, 4960–4964 (2011).

[13] Zheng Y (2019). An intrinsically stretchable high‐performance polymer semiconductor with low crystallinity. Adv. Funct. Mater..

[14] Zhang F (2013). Ultrathin film organic transistors: precise control of semiconductor thickness via spin-coating. Adv. Mater..

[15] Park S (2018). Self-powered ultra-flexible electronics via nano-grating-patterned organic photovoltaics. Nature.

[16] Park KS (2016). Inkjet-assisted nanotransfer printing for large-scale integrated nanopatterns of various single-crystal organic materials. Adv. Mater..

[17] Park JU (2007). High-resolution electrohydrodynamic jet printing. Nat. Mater..

[18] Pitsalidis C (2016). High mobility transistors based on electrospray-printed small-molecule/polymer semiconducting blends. J. Mater. Chem. C.

[19] Park KS (2013). Single-crystal organic nanowire electronics by direct printing from molecular solutions. Adv. Funct. Mater..

[20] Chen J-Y (2011). Manipulation on the morphology and electrical properties of aligned electrospun nanofibers of poly(3-hexylthiophene) for field-effect transistor applications. Macromolecules.

[21] Huang Y (2017). Hyper-stretchable self-powered sensors based on electrohydrodynamically printed, self-similar piezoelectric nano/microfibers. Nano Energy.

[22] Cho B (2014). Single-crystal poly(3,4-ethylenedioxythiophene) nanowires with ultrahigh conductivity. Nano Lett..

[23] Cui Y, Lieber CM (2001). Functional nanoscale electronic devices assembled using silicon nanowire building blocks. Science.

[24] Lee SW (2010). Periodic array of polyelectrolyte-gated organic transistors from electrospun poly(3-hexylthiophene) nanofibers. Nano Lett..

[25] Sun Y, Dong T, Yu L, Xu J, Chen K (2020). Planar growth, integration, and applications of semiconducting nanowires. Adv. Mater..

[26] Wang S (2012). Organic field-effect transistors based on highly ordered single polymer fibers. Adv. Mater..

[27] Wu Y, Su B, Jiang L, Heeger AJ (2013). “Liquid-liquid-solid”-type superoleophobic surfaces to pattern polymeric semiconductors towards high-quality organic field-effect transistors. Adv. Mater..

[28] Lin CJ (2011). High-performance FETs prepared from electrospun aligned P4TDPP nanofibers. Macromol. Chem. Phys..

[29] Lee S, Moon GD, Jeong U (2009). Continuous production of uniform poly(3-hexylthiophene) (P3HT) nanofibers by electrospinning and their electrical properties. J. Mater. Chem..

[30] Wang D (2018). Nanoscale coaxial focused electrohydrodynamic jet printing. Nanoscale.

[31] Xu Q (2013). Coaxial electrohydrodynamic atomization process for production of polymeric composite microspheres. Chem. Eng. Sci..

[32] Plog J, Jiang Y, Pan Y, Yarin AL (2021). Electrostatically-assisted direct ink writing for additive manufacturing. Addit. Manuf..

[33] Alenezi H, Cam ME, Edirisinghe M (2021). Core–sheath polymer nanofiber formation by the simultaneous application of rotation and pressure in a novel purpose-designed vessel. Appl. Phys. Rev..

[34] Jung EH (2019). Efficient, stable and scalable perovskite solar cells using poly(3-hexylthiophene). Nature.

[35] Yang, K. et al. Tunable flexible artificial synapses: a new path toward a wearable electronic system. *npj Flex. Electron.* **2** (2018).

[36] Wang L (2019). A Eu 3+ -Eu 2+ ion redox shuttle imparts operational durability to Pb-I perovskite solar cells. Science.

[37] Kim G (2020). Impact of strain relaxation on performance of α-formamidinium lead iodide perovskite solar cells. Science.

[38] Chabinyc ML (2008). X-ray scattering from films of semiconducting polymers. Polym. Rev..

[39] Rivnay J, Mannsfeld SC, Miller CE, Salleo A, Toney MF (2012). Quantitative determination of organic semiconductor microstructure from the molecular to device scale. Chem. Rev..

[40] Zhang T (2021). Superfast growth dynamics of high-quality silicon nanowires on polymer films via self-selected laser-droplet-heating. Nano Lett..

[41] Wei Z (2010). Organic single crystal field-effect transistors based on 6H-pyrrolo[3,2-b:4,5-b]bis[1,4]benzothiazine and its derivatives. Adv. Mater..

[42] Haiqing L, Reccius CH, Craighead HG (2005). Single electrospun regioregular poly„3-hexylthiophene nanofiber field-effect transistor. Appl. Phys. Lett..

