# Supplementary material for: High-performance flexible organic field effect transistors with print-based nanowires
Source: Microsyst Nanoeng. 2023 Jun 13;9:80. doi: 10.1038/s41378-023-00551-x (PMC10264417; doi:10.1038/s41378-023-00551-x)
Supplement: Supplementary file 1 — Revised Supplementary figures [file 41378_2023_551_MOESM1_ESM.pdf]

Supplementary Information

High-performance flexible organic field effect transistors with print-based nanowires

Liangkun Lu[1], Dazhi Wang[1, 3, 4*], Changchang Pu[1], Yanyan Cao[2], Yikang Li[1], Pengfei Xu[1], Xiangji Chen[1], Chang Liu[1], Shiwen Liang[4], Liujia Suo[1], Yan Cui[1], Zhiyuan Zhao[2*], Yunlong Guo[2], Junsheng Liang[1], Yunqi Liu[2]

Supplementary Figures

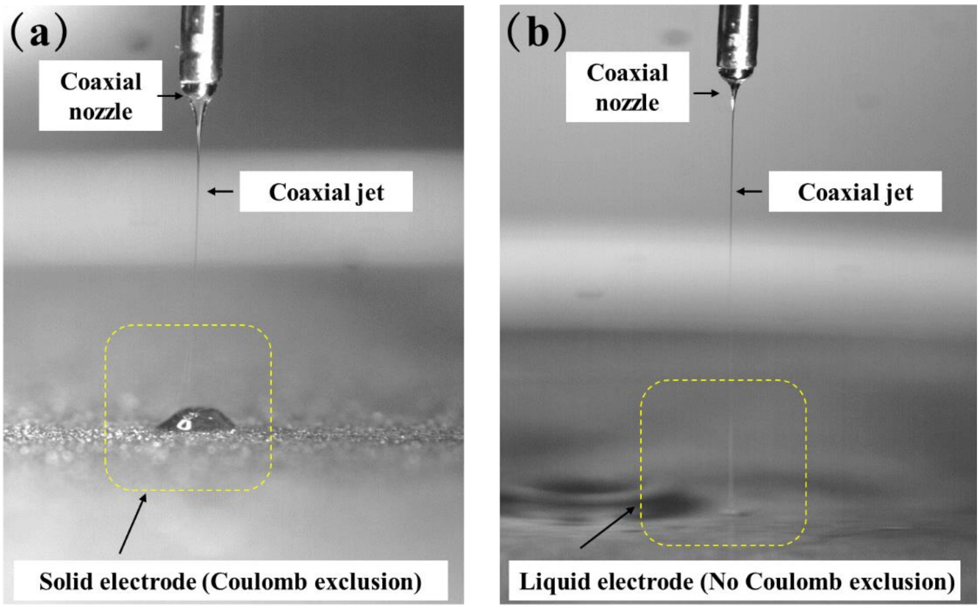

Supplementary Fig. 1 a Image of CFEJ printing process with metal electrode. **b** Image of CFEJ printing process with metal-liquid composite electrodes.

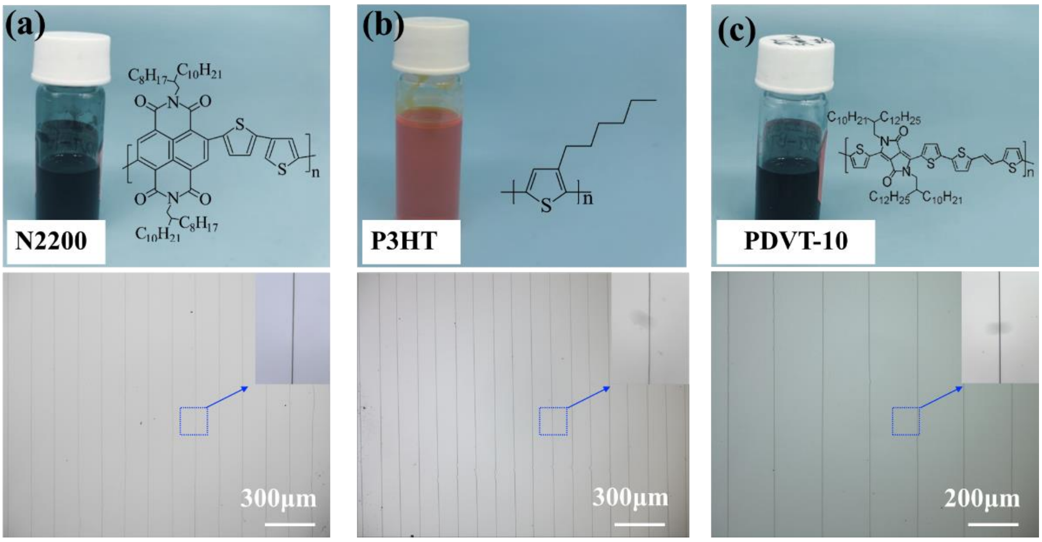

Supplementary Fig. 2 a-c Linear array structures of N2200 polymer ink. **d-f** Linear array structures of P3HT polymer ink. **g-h** Linear array structures of PDVT-10 polymer ink.

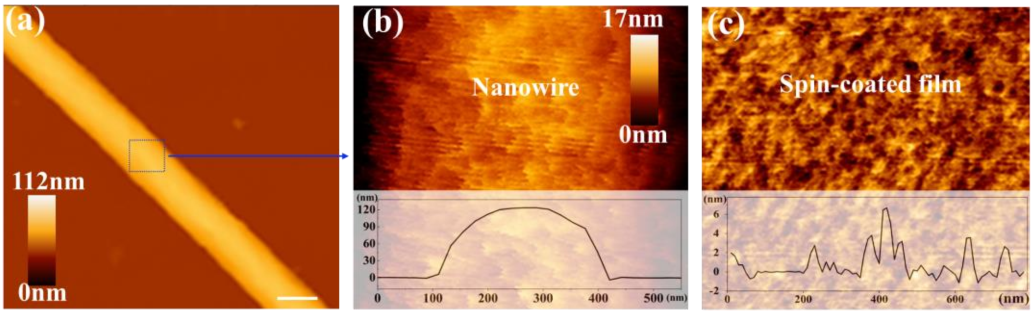

Supplementary Fig. 3 AFM image of sub-microwire and spin-coated thin film. **a,b** AFM of single IDT-BT nanowire. **c** AFM image of spin-coated IDT-BT thin film.

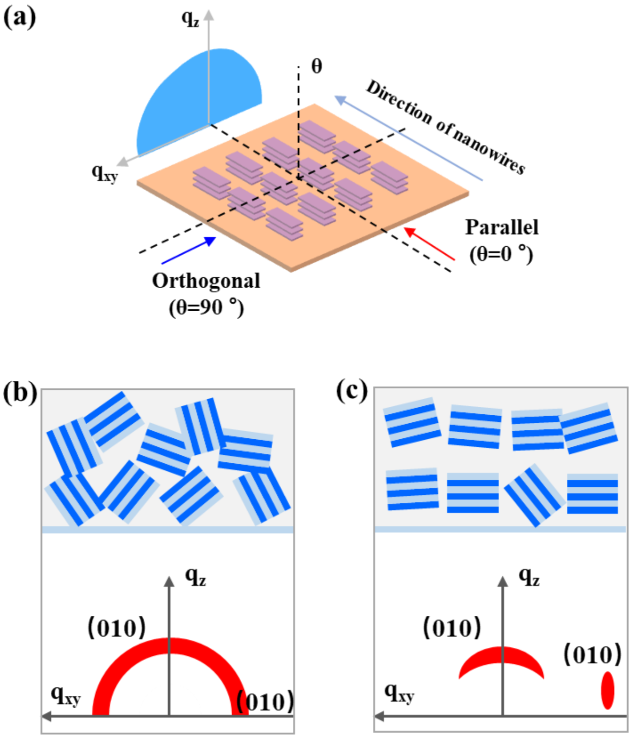

Supplementary Fig. 4 Correspondence between diffraction peaks and texture. (a) Randomly oriented arrangements of crystallites. (b) Textured with a distribution of crystallite orientations produce arcs of diffracted intensity.

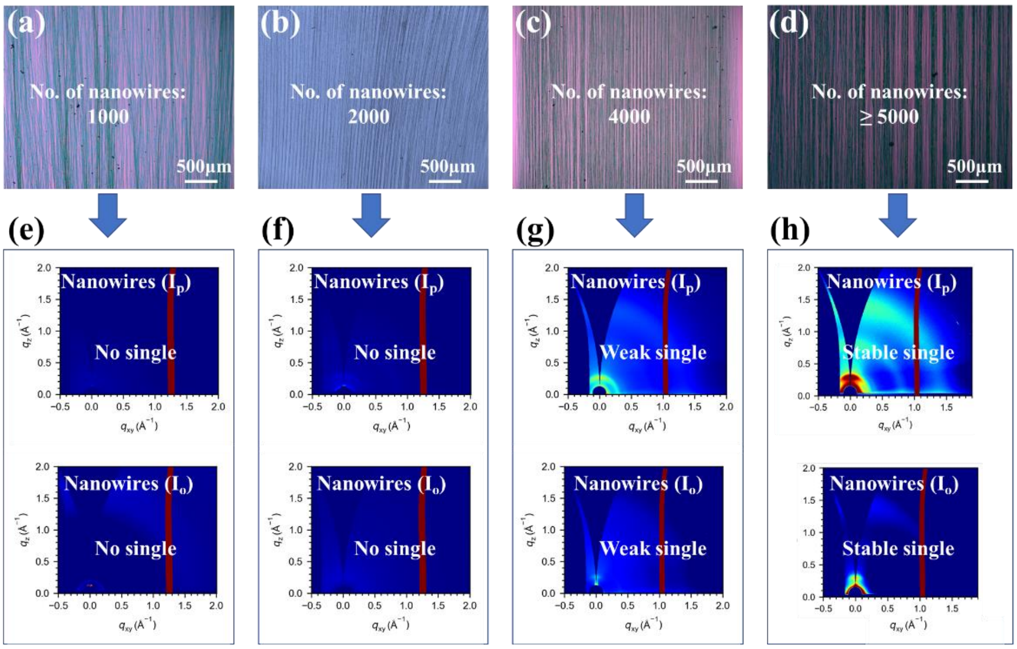

Supplementary Fig. 5 Relation between number of nanowire and diffraction peak strength

a,e (1000). **b,f** (2000). **c,g** (4000). **d,h** (⩾5000).

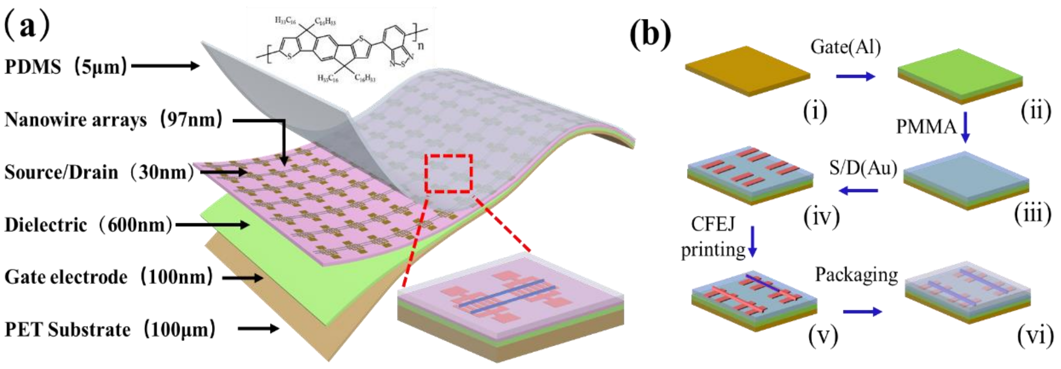

Supplementary Fig. 6 IDT-BT polymer NW array-based OFETs. **a** Bottom-gate bottom-contact device structure for flexible FETs. **b** Process diagram for NW array-based OFETs.

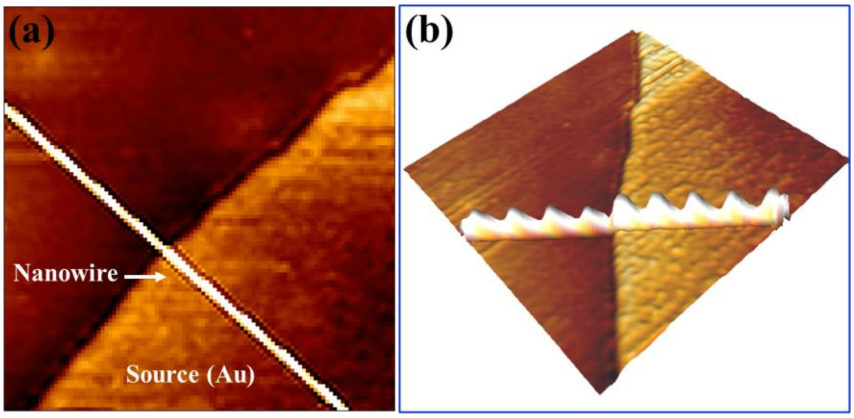

Supplementary Fig. 7 a AFM images of a NWs based OFET with source/drain electrodes. The images show the well-aligned IDTBT NW array channel between the Au electrodes**. b** 3-D AFM images of NWs lay on source/drain electrodes.

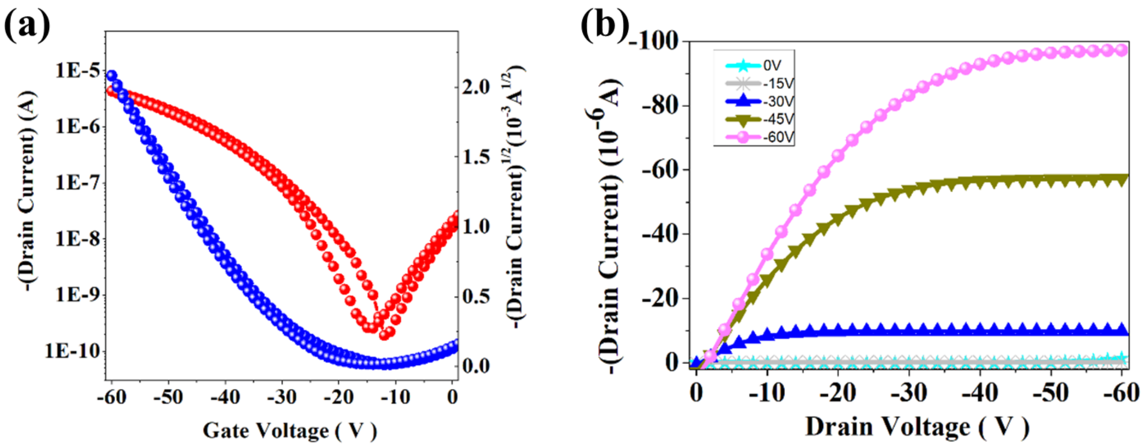

Supplementary Fig. 8 Device performance characteristics of IDT-BT thin film based OFETs.

a Transfer characteristic for the IDTBT film OFETs operated at a constant drain of -60 V. **b** Output characteristic for the IDTBT film OFETs operated at a constant drain of -60 V.

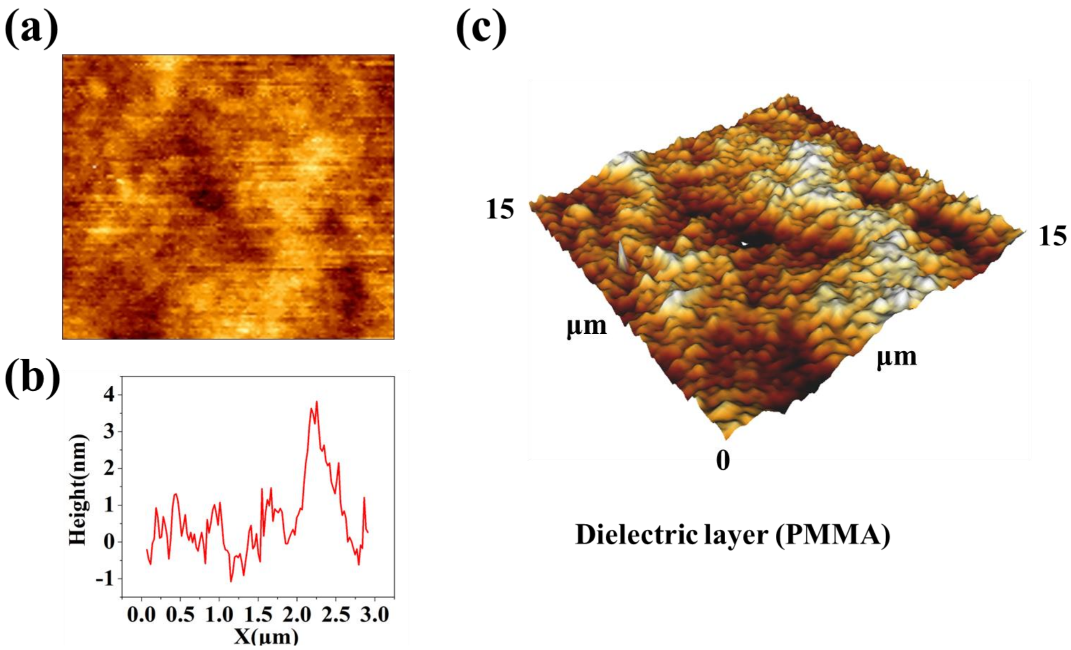

Supplementary Fig. 9 AFM image of spin-coated PMMA. **a,b** AFM image and roughness of PMMA. **c** 3-D AFM image of PMMA, the root-mean-square roughness values of PMMA were found to be 0.7 nm
